# Gli promotes tumor progression through regulating epithelial-mesenchymal transition in non–small-cell lung cancer

**DOI:** 10.1186/s13019-020-1049-x

**Published:** 2020-01-13

**Authors:** Long Jiang, Jia Huang, Yingjie Hu, Peiji Lu, Qingquan Luo, Lei Wang

**Affiliations:** 1 Shanghai Lung Cancer Center, Shanghai Chest Hospital, Shanghai Jiaotong University, Shanghai, 200030 China; 2 Department of Thoracic Surgery, Fourth Hospital of Hebei Medical University, Hebei, 050011 China

**Keywords:** Gli, Epithelial-Mesenchymal transition, Non–small-cell lung Cancer

## Abstract

**Introduction:**

Lung cancer is the leading causes of cancer-related deaths globally. The most frequent histologic type of lung cancer is non–small-cell lung cancer (NSCLC). NSCLC often undergo epithelial-mesenchymal transition (EMT). The components that control this process are thus promising therapeutic targets.

**Materials and methods:**

Gli/EMT protein expression levels were examined by western blot in paired NSCLC patient tissues and NSCLC cell lines. Functional analyses were performed to investigate SHH/Gli signaling and EMT in NSCLC cell lines. MTS cell viability, luciferase reporter, and western blot assays were performed to analyze pathway activity, while wound healing and transwell assays were executed to measure cell migration and invasion.

**Results:**

Higher Gli1 expressions were detected in tumor samples than in paired normal tissues. Differential expression of EMT biomarkers and activation of p-AKT were observed in tumor tissues. N-Shh stimulation of cells significantly increased reporter activity in NSCLC cell lines, while Gli-i treatment of transfected cells showed less relative reporter activity. When subjected to both Gli-i and N-Shh treatment, NSCLC cell lines continued to demonstrate decreased Gli transcriptional activity. Gli inhibition is associated with decreased expression level of p-AKT, N-cadherin and Vimentin. Knockdown of both Gli1 and Gli2 showed decreased EMT, migrative and invasive ability. Cells stimulated by N-Shh demonstrated greater mobility. In addition, AKT-i treated cells also demonstrated inhibited EMT activity.

**Conclusions:**

This study provides evidence for aberrant upregulation of the Gli signaling pathway and a strong association between expression of Gli versus AKT and EMT markers in NSCLC.

## Background

Lung cancer, one significant health problem worldwide, representing the most frequent cause of cancer mortality in many countries, and accounting for approximate 1.6 million deaths each year [1]. Only about 4–17% of patients with clinically detectable lung cancer could survival over 5 years depending on stage and regional differences, mainly due to lacking an effective and sensitive tool for early detection [2]. Therefore, steps in the right direction are needed at earlier detection and better treatment management of lung cancer patients.

Recently, various cell subpopulations, containing different gene or protein profiles, are identified by immunohistochemistry and comprehensive high-throughput platform [3]. Indeed, cellular plasticity could enhance such heterogeneity [4]. The majority of malignances arise from epithelial tissues [5]. A main characteristic of epithelial cells is its strong intercellular adhesion with constraint of cell mobility [6]. Nevertheless, the epithelial feature of epithelial cells can be able to shed through epithelial-to-mesenchymal transition (EMT) [4]. Under this circumstance, some cells may experience oncogenic EMT, which means loss of cell–cell adhesion and polarity, tissue organization, and showing phenotypic conversion from epithelial cells into mesenchymal-like cells, enhancing migratory and invasive capacity, promoting early stage dissemination of cancer cells from the local primary tumor, and inhibiting apoptosis and senescence. The EMT process establishes colonization and progression of tumor cells in metastatic sites, which has been identified as a critical event initiating tumor invasion and metastasis in vitro and in vivo.

EMT malignant cells could enable the access to the metastatic site of non-EMT cells, resulting in macro-metastatic initiation [4]. Thus, metastasis procedure can be influenced by intratumoral heterogeneity: where neighboring non-EMT tumor cells might be affected by a small number of primary EMT tumor cells. It is generally considered that EMT and metastasis are late-stage events in tumorigenesis. After acquiring the mesenchymal phenotype, the epithelial cell could detach from the primary site. EMT program activation and its potential association with metastasis may be a significant event in cancer progression. According to the traditional hypothesis, a small amount of cancer cells in close contact with microenvironmental stroma may acquire EMT ability after receiving inducing signals.

Sonic Hedgehog (SHH) pathway has been implicated to acting through several components to activate the GLI zinc-finger transcription factors [7]. The signaling pathway is composed of 3 SHH ligands [8]. Canonical activation of Hh/Gli pathway in mammals includes binding of the Hh ligands to patched receptors. When SHH ligand is absent, the transmembrane protein smoothened would be inhibited by patched receptors [9]. In the presence of SHH ligand, patched receptors would be engaged. Followed by diminished inhibitory by patched receptors, smoothened would be activated. The signal would then be transduced to the downstream transcription factors GLI1, GLI2, and GLI3 [10]. When inhibition is released, the GLI transcription factors would increase in the nucleus, then activating Hh target genes [11]. The SHH signaling act as a primary regulator in controlling a series of developmental processes [12].

In the current study, abnormal upregulation of the Gli signaling was observed in both lung cancer tissues and cell lines. Associations between expression of Gli and AKT and EMT markers were identified in lung cancer specimens. Gli inhibition is linked to decreased p-AKT expression and EMT activity. Inhibition of AKT and Gli signal pathway would result in decreased EMT, both of which are associated with greater aggression and viability in NSCLC.

## Methods

### Cell culture and treatment

Human NSCLC cellsA549 and H1975 were adopted from American Type Culture Collections (Manassas, VA). The Gli1 siRNA, Gli2 siRNA, and control siRNA were from Cell Signaling (Danvers, MA). GANT61, the selective inhibitor of Gli transcription in the Hedgehog pathway, was commercially purchased (Selleckchem, Munich, Germany). All cell lines were cultured with indication from American Type Culture Collections. Cells were plated and transfected with siRNA using Lipofectamine RNAiMAX (Invitrogen, Carlsbad, CA) according to the manufacturer’s protocol. Knockdown mediated by siRNA was confirmed by Western blot and RT-PCR. Cells were harvested for preparing assays 48 h after transfection. DMSO was used for dissolving CAY10561 and FR180204.

### Luciferase reporter assay

The luciferase plasmid (Promega, Madison, WI) were transfected using Lipofectamine RNAiMAX or Lipofectamine 2000 (Invitrogen, Carlsbad, CA), depending on the manufacturer’s protocol. After 48 h, cells were harvested using a Dual-Luciferase Reporter Assay Kit (Promega, Madison, WI) in a 96-well plate. According to instructions applied, detection was conducted on a GloMax-96 Microplate Luminometer (Promega, Madison, WI).

### Western blotting

Extraction of total protein from cell lines was performed by M-PER Mammalian Protein Extraction Reagent (Thermo, San Jose, CA) and Complete Protease Inhibitor Cocktails (Roche, Lewes, UK), following the manufactures’ protocols. The concentrations of protein were determined using Pierce BCA Protein Assay Kit (Thermo, San Jose, CA). A total of 10 μg of proteins were run on SDS–polyacrylamide gels and transferred to Immobilon-P membranes (Millipore, Bellerica, MA), then blocked in 5% nonfat milk and incubated with the primary antibodies at 4 °C overnight. Then the membranes were probed with relevant secondary antibodies, followed by analysiswith an ECL blotting analysis system (Amersham Pharmacia Biotech, Piscataway, NJ).

### Cell viability assay

Cells lines were cultured and treated by GANT61 with dosage gradients. After incubated for 48 h, cells lines were lysed and analyzedin a CellTiter-Glo Luminescent Cell Viability Assay system (Promega, Madison, WI). Luminescent signaling was detected by the GloMax-96 Microplate Luminometer. Cell viability assay was performed by Vybrant® MTT Cell Viability Assay, by which IC50 and dose-response curves were calculated.

### Wound-healing assay

Cell lines were treated with DMSO (control), N-Shh (0.5 mg/mL), GANT61(15umol/L), or GANT61(15umol/L) + N-Shh (0.5 mg/mL). The wound-healing was conducted by a 200 μl pipette tip scratch after 48-h transfection. Then medium was replaced fresh and cells were cultured continuously. Images were taken with a microscope at the time of wound-healing (0 h) and then every 6 h. Wound-healing areas were measured using ImageJ software.

### Statistical analysis

Data were showed as mean ± standard deviation (SD) calculated from three independent repeats. All the statistical analyses were computed by the SPSS 22.0 for Windows software system (SPSS Inc., Chicago, IL). One-way analysis of variance (AVONA) was used for analyses among multiple groups. A significant difference was defined as the *P* value less than 0.05.

## Results

### In NSCLC tissue samples, Gli is upregulated and associated with AKT and EMT pathway markers

**Fig. 1:**
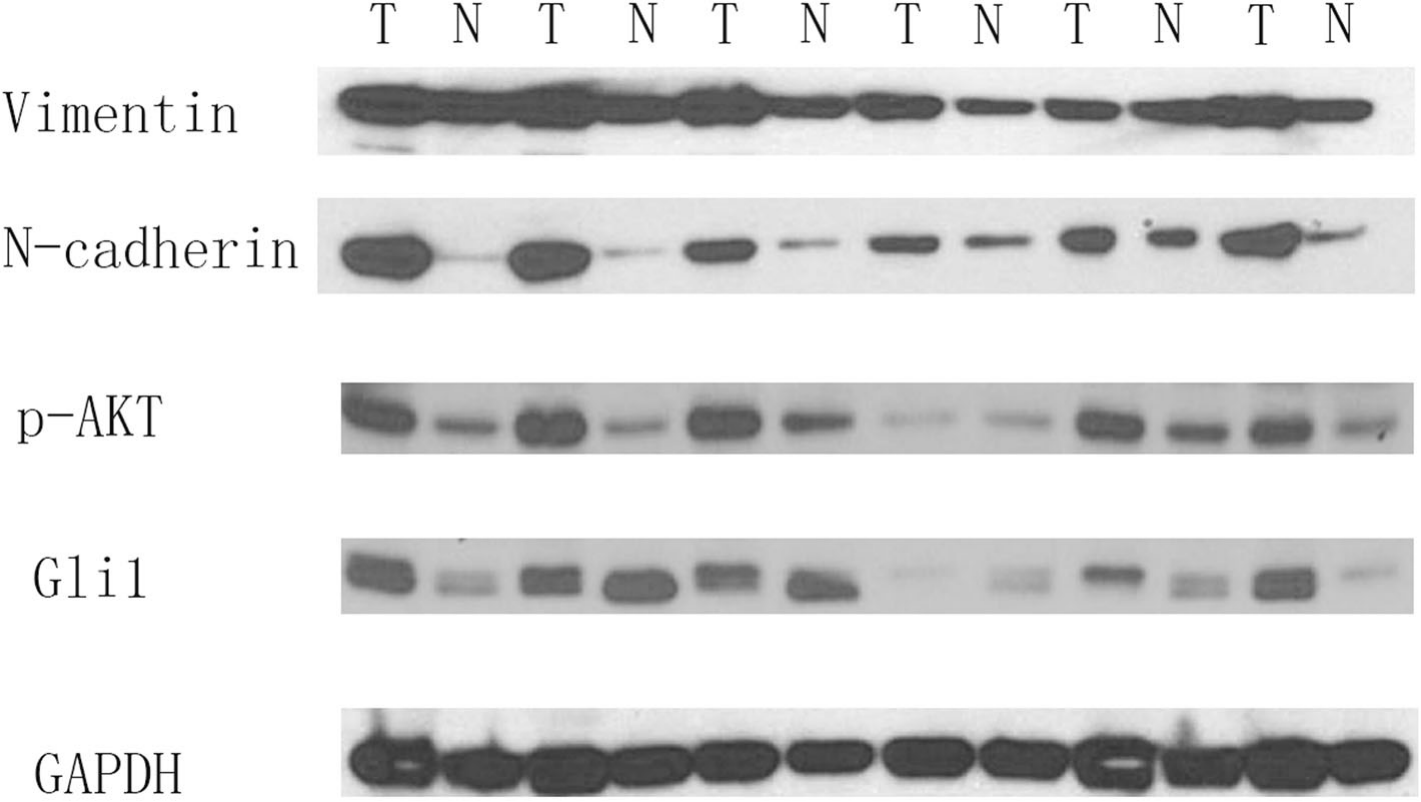
Gli1, p-AKT, and EMT pathway markers are upregulated in NSCLC tissue samples. Western blots of Gli1 protein expression in 36 matched pairs of NSCLC tumor (T) and normal (N) tissues. GAPDH served as a loading control; results of 6 representative sample pairs are shown here. p-AKT and EMT markers (N-cadherin and Vimentin) were examined. NSCLC: Non–small-cell lung cancer

Gli1, p-AKT, and EMT pathway markers was detected in 36 matched NSCLC and normal patient tissue samples in protein level. In western blot analyses, 61.1% (22/36) of cancer samples illustrated higher Gli1 expression level than in paired normal tissue samples. High expression level of N-cadherin, a biomarker indicating increased EMT, was examined in 72.2% (26/36) in cancer tissues. Overexpression of Vimentin, associated with EMT activation characteristics, was detected in 77.8% (28/36) of the tissue samples. Subsequently, proteins in AKT pathway were analyzed by western blot. Activation of p-AKT was observed in 75% (27/36) of the cancer tissues. Subsequent correlation analyses between Gli1 and EMT or AKT pathway markers showed positive correlation as 0.7774 (*p* < 0.001) of Gli1 and N-cadherin, 0.6701 (p < 0.001) of Gli1 and Vimentin, 0.7237 (*p* < 0.001) of Gli1 and p-AKT, respectively. As a result, our findings demonstrated Gli1 activation in cancer tissue samples, with significant correlations to EMT and AKT pathway markers (Fig. 1).

### Gli inhibition and siRNA knockdown reduces EMT, cell viability, and p-AKT expression in NSCLC cell lines

**Fig. 2:**
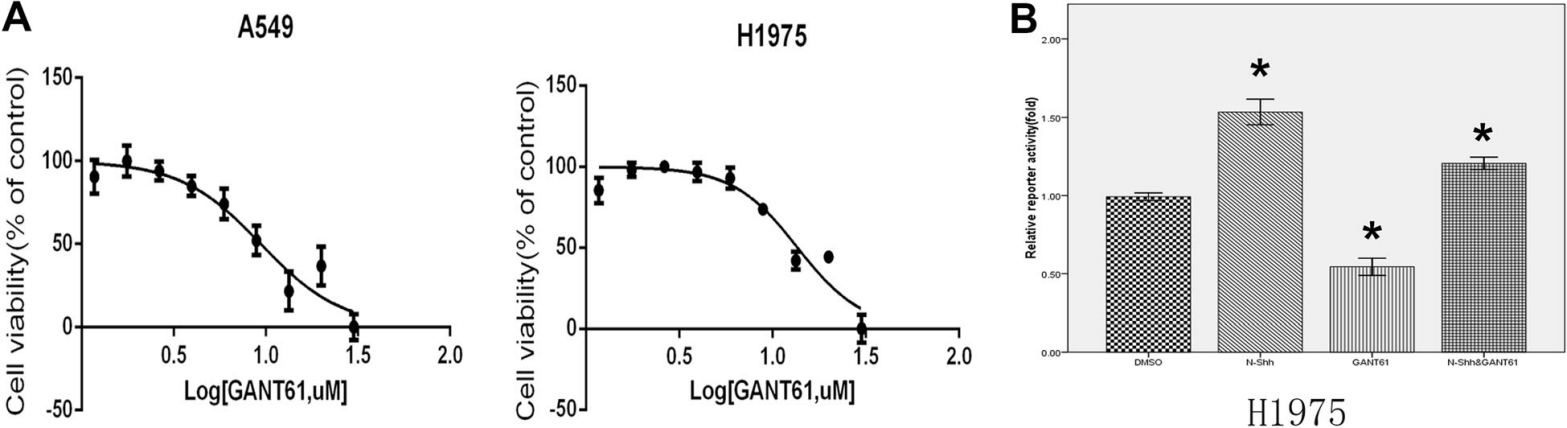
Gli inhibition reduces EMT activity in NSCLC cell lines. **a** MTS cell viability assay in A549 and H1975 NSCLC cell lines. Cells were subjected to a serial dilution of Gli-i with DMSO control over a 3-day period, yielding IC_50_ values of 9.385 nM and 13.61 nM, respectively. **b** Luciferase reporter assay in H1975 cell lines, treated with DMSO (control), N-Shh (0.5 mg/mL), GANT61(15umol/L), or GANT61(15umol/L) and N-Shh (0.5 mg/mL) stimulation, for 24 h. Results are expressed as fold activity, i.e. the ratio of luciferase activity induced in Gli-transfected cells relative to basal luciferase activity in control transfected H1975 cells (*p*-values of < 0.05 was indicated as *)

In order to explore the role of Gli in EMT, two NSCLC cell lines, A549 and H1975, were used in cell viability (Fig. 2a), and luciferase reporter assay (Fig. 2b). N-Shh was used to stimulate the cells.

MTS assays in both A549 and H1975 NSCLC cell lines, using Gli-i with DMSO as vehicle control, yielded IC50 values of 9.385uM and 13.61 nM, respectively (Fig. 2a). The result indicates that inhibition of Gli may remarkably reduce NSCLC cell viability. Luciferase reporter assays in these two cell lines were conducted to determine the transcriptional activity mediated by Gli. As expected, N-Shh stimulated cells drastically promote reporter activity in H1975 cell line (*p* < 0.05), while GANT61 treatment demonstrating approximately 50% less reporter activity. When subjected to both GANT61 and N-Shh treatment, H1975 cell line demonstrated increased Gli transcriptional activity (Fig. 2b).

**Fig. 3:**
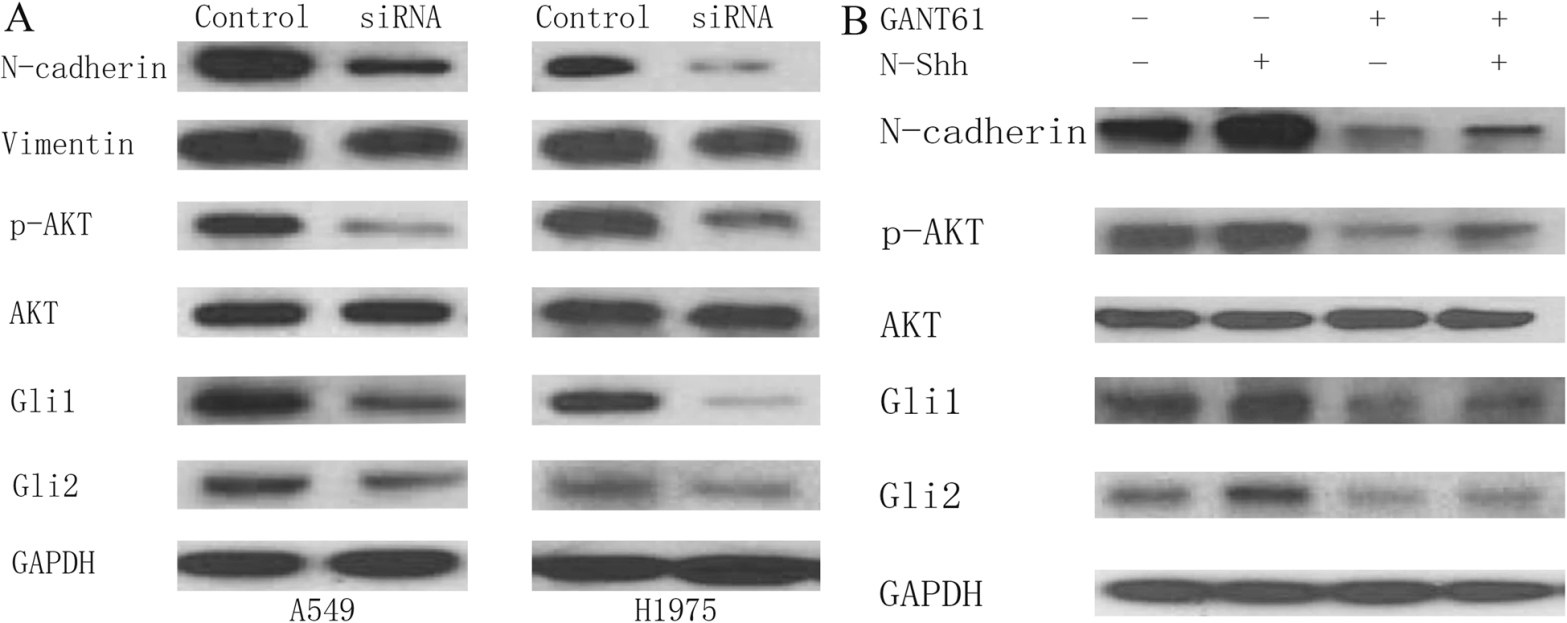
The effect of Gli signaling on EMT and p-AKT expression in NSCLC cell lines. **a** Western blots of A549 and H1975 cells, subjected to siRNA treatment. Cells were given control siRNA (100 nM) or Gli1 + Gli2 siRNA (100 nM each) for 48 h prior to total protein extraction. Expression of Gli1, Gli2, N-cadherin, Vimentin, p-AKT and AKT expression were examined, with GAPDH serving as an internal control. **b** Western blots of H1975 cells, treated with DMSO (control), N-Shh (0.5 mg/mL), GANT61(15umol/L), or GANT61(15umol/L) and N-Shh (0.5 mg/mL) stimulation, for 24 h prior to total protein extraction. Expression of Gli1, Gli2, N-cadherin, p-AKT and AKT expression were examined, with GAPDH serving as an internal control

To explore protein expression of EMT and relevant markers after siRNA treatment (Gli1 + Gli2 siRNA), western blots were performed in A549 and H1975 (Fig. 3a). siRNA knockdown of Gli is linked to lower p-AKT expression level. siRNA knockdown of Gli also results in a decrease in N-cadherin and Vimentin. Additionally, as shown in Fig. 3b, in H1975 cell lines, GANT61 treatment inhibited EMT activity (decreased N-cadherin). Cells stimulated by N-Shh demonstrated increased EMT activity (increased N-cadherin), even still less than the blank control when administered GANT61. p-AKT expression was also inhibited by GANT61 administration, whereas promoted by N-Shh stimulation. In total, these data obviously indicate that Gli signaling pathway inhibition may suppress EMT, cell viability, and p-AKT expression in NSCLC.

### Gli signal pathway induces cell migrative and invasiveactivity in vitro

**Fig. 4:**
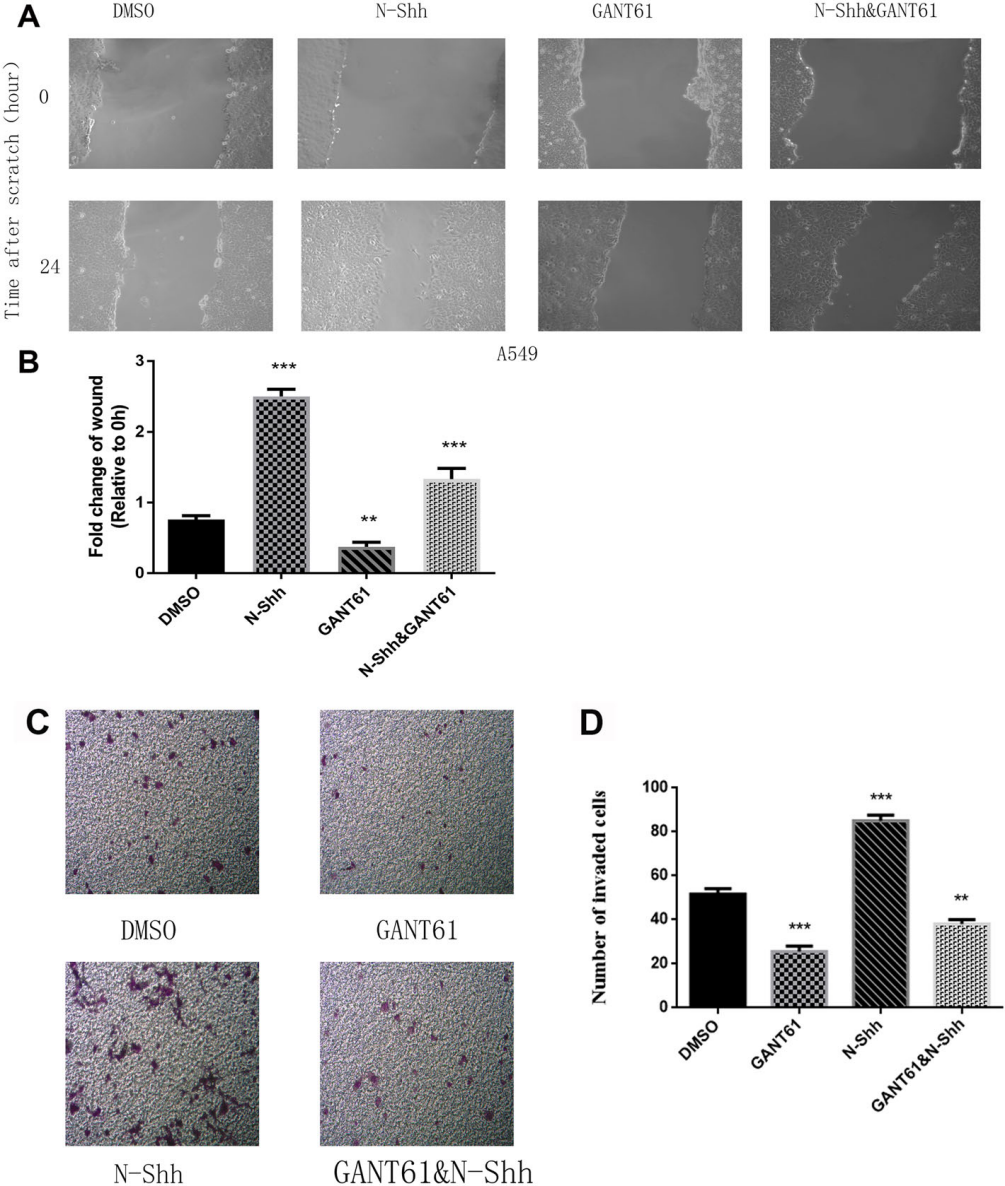
Gli signaling promotes cell migration and invasion in NSCLC cell lines. **a** Wound healing assays of A549 lung cancer cells, treated with DMSO (control), N-Shh (0.5 mg/mL), GANT61(15umol/L), or GANT61(15umol/L) with N-Shh (0.5 mg/mL) stimulation. Representative images captured by a light microscope are shown (100x). **b** Quantification of wound healing assays, expressed as percentage of wound in comparison to 0 h (*p*-values of < 0.01 or < 0.001 were indicated as ** or ***, respectively). **c** Transwell invasion assay of A549 lung cancer cells, treated with DMSO (control), N-Shh (0.5 mg/mL), GANT61(15umol/L), or GANT61(15umol/L) with N-Shh (0.5 mg/mL) stimulation. Representative images captured by a light microscope are shown (100x), average of 5 picture fields at 100x total magnification. **d** Quantification of transwell invasion assays, expressed as percentage of wound in comparison to 0 h (*p*-values of < 0.01 or < 0.001 were indicated as ** or ***, respectively)

To evaluate the role of Gli signal pathway on migrative and invasive activity, wound healing (Fig. 4a & b) and transwell assays (Fig. 4c & d) were performed in A549 cells, under the treatment with DMSO (vehicle control), N-Shh, GANT61, and GANT61 + N-Shh as described above. After 24-h treatment of GANT61, A549 cells exhibited significantly slower migration. Cells treated by N-Shh illustrated greater invasive activity, while cells migrated slower than GANT61 treatment when the administered the DMSO control (Fig. 4a & b). Moreover, in invasive activity assessment, A549 cell lines illustrated inhibited invasive ability after treated by GANT61. Cells administered N-Shh showed increased invasive ability, also such cells invaded faster than GANT61 treatment compared with the DMSO control (Fig. 4c & d).

### Inhibition of AKT inhibits cell invasive activity in vitro

**Fig. 5:**
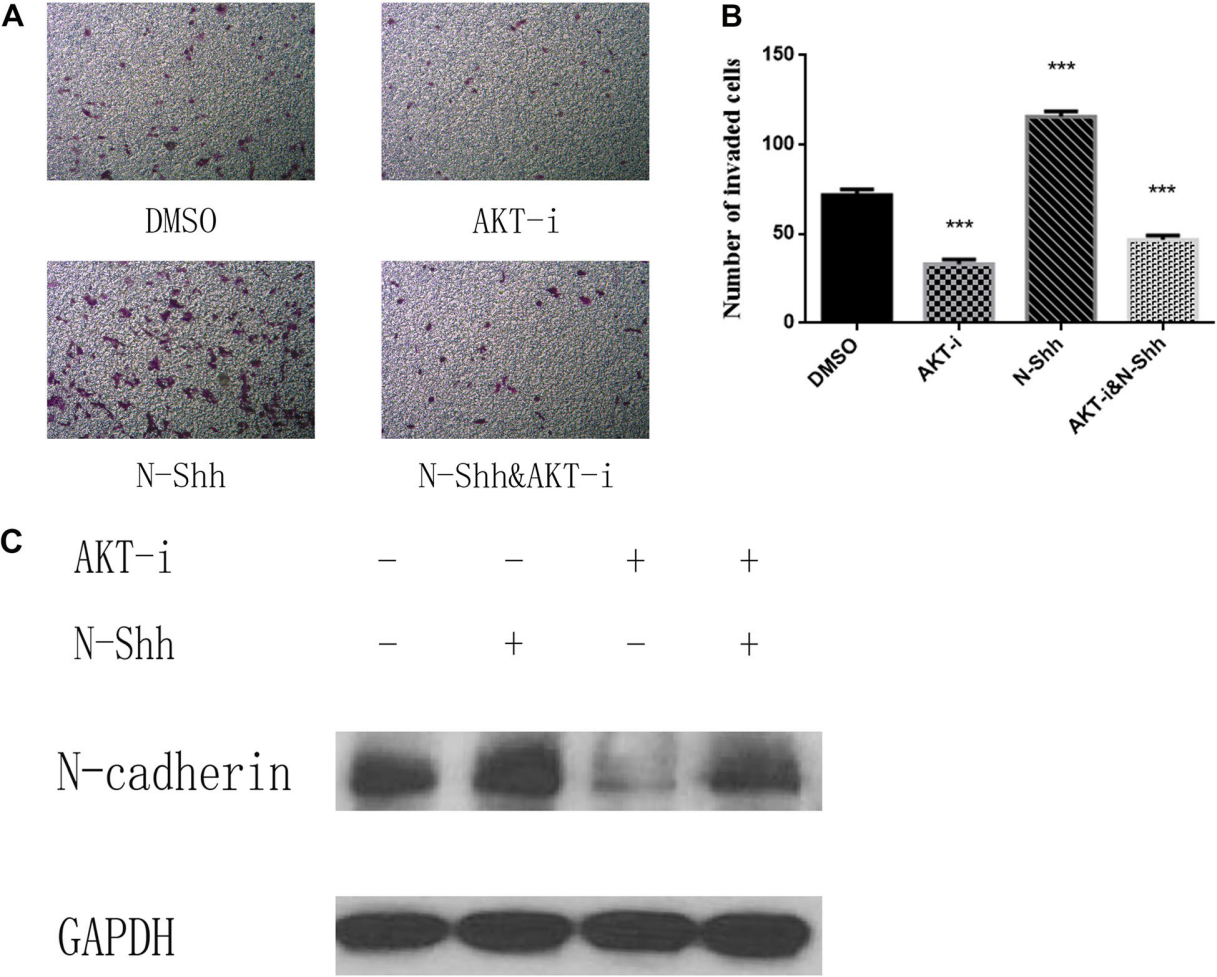
AKT inhibition reduces cell invasion and EMT in NSCLC cell lines. **a** Transwell invasion assay of H1975 lung cancer cells, treated with DMSO (control), N-Shh (0.5 mg/mL), AKT-i(1 μmol/L), or AKT-i(1 μmol/L) with N-Shh (0.5 mg/mL) stimulation. Representative images captured by a light microscope are shown (100x), average of 5 picture fields at 100x total magnification. **b** Quantification of transwell invasion assays, expressed as percentage of wound in comparison to 0 h (*p*-values of < 0.001 was indicated as ***). **c** Western blots of EMT marker was examined in H1975 (N-cadherin), with GAPDH as a loading control. Cells were pretreated with DMSO, N-Shh (0.5 mg/mL), AKT-i(1 μmol/L), or AKT-i(1 μmol/L) with N-Shh (0.5 mg/mL) stimulation prior to protein extraction

To evaluated the role of AKT signal pathway on cell invasion in NSCLC, H1975 cell lines were subjected to treatment with DMSO, N-Shh (0.5 mg/mL), AKT inhibitor MK-2206 2HCI (1umol/L), AKT inhibitor MK-2206 2HCI(1umol/L) for 30 min, then administrated N-Shh (0.5 mg/mL) for 24 h (Fig. 5a & b). After 24-h treatment, H1975 cells invaded significantly slower when administrated AKT inhibitor MK-2206 2HCI. Cells treated with N-Shh exhibited increased invasive ability, while they invaded faster than AKT inhibitor MK-2206 2HCI compared with the DMSO control.

### AKT inhibition reduces EMT in NSCLC cell lines

Subsequently, further experiments on AKT signal pathway was performed. H1975 cell lines were treated with N-Shh, an AKT inhibitor (AKT-i) MK-2206 2HCI. After that, western blot analysis was performed to determine relevant pathway and EMT markers in protein levels after 30-h treatment (Fig. 5c). In H1975 cell lines, AKT-i treated cells inhibited EMT activity (decreased N-cadherin). Thus, AKT and Gli signal pathway inhibition might result in decreased EMT, associating with greater aggressive ability in NSCLC.

## Discussion

Because of the advanced lung cancer presented at the time of diagnosis, few if any available interventional options for effective therapies were left [3]. Therefore, tumor invasion and metastasis constitute critical steps in the pathogeneses of lung cancer, the understanding of which may provide novel molecular insight in tumor progressive and metastatic mechanisms and ultimately yield novel molecular-targeted therapeutic strategies [4].

SHH/Gli signaling pathway is significant in carcinomas; following effectors are linked to EMT, and may serve as an potential therapeutic target of many types of cancers [13]. The current study offers preliminary evidence of in vitro Gli inhibition decreases the migrative and invasive abilities of NSCLC cells by reducing EMT. Lung cancer patients tissues illustrated positive associations between Gli1 and EMT markers, including N-cadherin and Vimentin, both of which are associated with increased EMT [14]. Data also demonstrated phosphorylated AKT associating with tumorigenesis and aggression [15]. After showing that elevated levels of SHH/Gli were associated with EMT and relative regulators, functional studies indicate the remarkable effects of inhibited Gli on impairing their expression. Novel Gli-i small molecular compound, in addition to siRNA and AKT-i inhibition, would result in decreased protein levels involving EMT. These phenomena are supported by wound healing and invasive assays. Study via western blot showed a comprehensive overview of potential mechanisms of inhibited migrative and invasive abilities following inhibition of SHH signal pathway.

While recognized as critical factors in biological development, SHH signal pathway has rapidly underwent intense study in oncology [8]. Significantly, interaction between tumor cells and the tumor microenvironment was mediated by Hh signaling, which might result in proliferation and metastasis in multiple tumor types [9]. In other words, the SHH pathway is implicated in several types of cancers, such as basal cell carcinomas, medulloblastomas, gliomas, sarcomas, and pancreatic carcinomas. Although many cancers activate the pathway either by activation of smoothened or inactivation of patched, some tumors involve in SHH signaling by increasing the expression levels of GLI, such as rhabdomyosarcoma, osteosarcoma, glioma, breast cancer, pericytoma, prostate cancer, and Ewing sarcoma family of tumors. Therefore, GLI would be a potential beneficial therapeutic target for a wide spectrum of cancer patients.

EMT-inducing transcription factors, such as Twist1, Snail1, ZEB and Six1, have been proved to be associated with metastasis by experiments involving loss-of-function and gain-of-function about EMT [16]. For example, E-cadherin exhibits the ability of promoting cell adhesion and preventing tumor invasion and metastasis [17]. Moreover, Twist1 has been proved the role of enhancing metastasis in many types of cancers [18]. These data demonstrated that induction of EMT may induce tumor metastatic progression in vivo. In response to EMT-inducing pathway, the expression level of epithelial cell markers is down-regulated in epithelial cells. By contrast, the mesenchymal markers are up regulated. Pivotal functional processes, such as cell proliferation, migration and invasion, could be regulated by EMT-inducing transcription factors [17]. Moreover, EMT-inducing transcription factors are downregulated post embryogenesis, but re-expressed when carcinogenesis, which would result in increased tumor initiation and enhanced metastasis. In tumor cells, Twist1 and Snail1 could repress E-Cadherin and upregulate mesenchymal genes [4].

EMT regulation is a complicated process; the SHH/Gli pathway may take part in at different levels [7], thus, the detailed mechanism involving SHH signaling regulating EMT process in lung cancer needs further investigation. Considering cell lines used in the current study, some reports have suggested that H1299 cell line might be a more suitable tool in EMT research. However, H1975, which was selected in the current study, has also been widely used in many other studies.

During carcinogenesis, progression and metastasis, EMT act together with other key signaling pathways, such as Wnt, SHH, and so on [10]. Till now, there is no direct evidence linking SHH networks to lung cancer in the aspect of EMT. Furthermore, Shh signaling has been found to be essential for lung cancer onset and progression [11]. Consistently, Twist1 is intimately associated with Hh/GLI signaling pathway during normal and disease-related procedures [19]. Recently, Twist1 and Snail1 were proved to take part in the Hh signaling in tumor-initiating cells [20]. EMT transcription factors Twist1, Snail1 and Six1 would influence carcinoma cells by inducing EMT characteristics and aggressive properties [21]. EMT transcription factors activate GLI by employing different mechanisms, including Hh ligand depended signaling [22].

While further study is still necessary, Gli inhibition is a potential field deserving further study and promising drug development, because of its close association with EMT and regulation of tumorigenesis and aggression in lung cancer.

## Conclusion

The current study presents data for abnormal overexpression of the Gli pathway and a tight link between Gli versus AKT and EMT expression in lung cancer. The current results indicate that activation of SHH/Gli signaling pathway may be important in increasing cell survival and progression. Inhibiting of Gli and AKT pathway may thus serve as a potential therapeutic target for lung cancer patients.

## Data Availability

The analyzed data sets generated during the study are available from the corresponding author on a reasonable request.

## Abbreviation

AKT-i: AKT inhibitor
AVONA: Analysis of variance
EMT: Epithelial-mesenchymal transition
NSCLC: Non–small-cell lung cancer
SD: Standard deviation
SHH: Sonic Hedgehog
